# Seven Steps for Emergency Physicians to Dismantle Access Barriers and Build Equitable Care Systems

**DOI:** 10.5811/westjem.52395

**Published:** 2025-12-23

**Authors:** Alicia M. Gonzalez, Elizabeth Keating, Aimee Moulin

**Affiliations:** * University of California Davis Health, Department of Emergency Medicine, Davis, California; † University of California Davis Health, Department of Psychiatry, Davis, California; ‡ Public Health Institute, The Bridge Center, Oakland, California; § Pioneers Memorial Hospital, Brawley, California

As many as one fifth of the people who visit emergency departments (ED) annually report having no other source of healthcare, particularly in regions with primary care shortages. Since 2018, the Bridge Center at the Public Health Institute (Bridge) has led a national movement, shifting how EDs in hundreds of hospitals across more than 45 states address public health crises for populations historically underserved by the healthcare system. Change is hard; change in healthcare is particularly difficult. To address this gap, we developed a change-making framework that guides champions to disrupt the status quo and meaningfully improve the quality of care for patients who have few options beyond the ED. This versatile framework is based on implementation research and has been tested and shown to be successful at hundreds of hospitals where it has been applied to the treatment of substance use disorder, sexual and reproductive health needs, and harm reduction efforts including naloxone and emergency contraception distribution. Any champion can use this framework to decrease pre-existing bias, implement evidence-based practices for stigmatized health conditions, and create sustainable reform in US EDs and other acute care settings.

## INTRODUCTION

Hospital operations in 2026 have grown increasingly complex and often lack clear direction and coordination. Healthcare leaders are struggling to balance budgets with cuts to Medicare/Medicaid, facing a historic staffing shortage,^4^ and responding to ever-changing threats to public health. Nearly half of Americans are uninsured, underinsured, or burdened by medical debt.^5^ It’s getting worse, and disruption is needed. While bedside clinicians often have ideas for practical, patient-centered solutions, they often lack training to learn how to effectuate new ideas. This prevents clinical teams from making real-time changes. It can take up to 17 years for medical practice to catch up to evidence,^6^ leaving hospitals, clinicians, and patients frustrated.

The Bridge Center at the Public Health Institute (Bridge) has spent over six years testing, refining, and implementing our healthcare change-maker framework that has transformed the healthcare status quo in one-third the traditional time. Together, we hold decades of experience across clinical and policy spaces, driving physician-led reform. Here we present our novel change-making framework for the first time. Importantly, our experience centers around practice reform in historically stigmatized care, such as addiction treatment and reproductive health, including abortion. Much existing implementation science assumes that practice will follow the evidence, but in our experience, when leading change that relates to marginalized patients, additional work is needed to normalize why and how to care for those who have fallen through the cracks in the safety net. Our framework was originally designed for acute care settings, but it has proven to be effective in a variety of healthcare settings. Our work extends diffusion theory^7^ by investigating how existing scientific evidence can inform care delivery in settings where change-makers face institutional inertia or political sensitivity.

### Bridge Has Rapidly Transformed the Status Quo

Bridge was launched through its foundational CA Bridge program. California’s Department of Health Care Services funded CA Bridge to provide grants to hospitals to build medication for addiction treatment (MAT) programs. MAT—specifically a drug called buprenorphine—is the gold standard treatment^8^ for opioid use disorder but was and still is dramatically under-prescribed due to factors including stigma and incomplete medical training.^10^ Over five years, Bridge administered grants and/or technical assistance to 291 of California’s 330 hospitals to build MAT programs. This effort tracked over half a million patient navigator encounters and over 125,000 buprenorphine initiations. Bridge has also supported MAT program initiation in hospitals across 40 states, changed national standards for prehospital addiction treatment,^11^ and expanded access to sexually transmitted infection screening and reproductive healthcare.

Since its founding, Bridge has engaged hundreds of driven and compassionate clinical champions. Bridge clinical leaders and staff coach these champions to initiate practice changes by winning over hearts and minds, engaging multidisciplinary leadership teams, adding new medications to hospital formularies, embedding workflows in electronic health records, and training peers to use simple treatment protocols covering critical knowledge gaps historically missing from clinical training.

### Getting Started: Bust Through Bureaucracy to Build the Case for Change

Complex, overlapping reporting structures are often a barrier to clinician-led change. Most hospitals are led by a CEO who reports to a board of directors and is supported by a “C-Suite” that oversees the hospital’s medical care and operations. While clinicians can either be employees or contractors, staffing privileges are typically handled by the Medical Staff Office. The Chief of Staff, medical department chairs, and other physician leaders’ roles are also critical to understand, as are the multidisciplinary hospital committees and work groups that manage hospital policies, medical staff bylaws, and pharmacy formularies. Considering this complex organizational chart, champions must consider: Who has a stake in the process you’re trying to improve? Is this change replacing a current process, or is it building guidance where none exists? Does it require executive support, a literature review of scientific evidence, or support from a specific stakeholder?

Champions should begin by meeting with leaders to understand the hospital’s existing structure for change. Accomplishing this task may not be straightforward. Tips to get started:

1. *Ensure your immediate supervisor is on board*. Blindsiding your direct supervisor tends to result in more harm than good. You do not need to have your boss’s full support, but it’s tough to succeed if they are in direct opposition to your idea.
2. *Meet people in person when possible*. Many hospital leaders are physically present in the hospital during weekday business hours. Introducing yourself in person can go a long way in moving the conversation forward.
3. *Get to know the person who sends meeting invites and sets the agenda*. Clinical leaders are often busy and inconsistent in responding to e-mails or calls. Most committees or department meetings are supported by staff with more predictable availability.
4. *Expect to be told no, and don’t accept the first no*. We can’t tell you how many times the first three answers are “no” when advocating for practice change, particularly for stigmatized conditions such as opioid use disorder or pregnancy loss. Don’t let that deter you. “No” is far more common than “yes” when new ideas get pitched.

## FOLLOW THE TRAFFIC LIGHT ANALOGY

As you meet and discuss your idea with others, they will sort themselves into three “traffic light” categories based on their level of interest in or resistance to your idea: green; yellow; and red lights.

### First: Identify and Engage Green Lights

“Green lights” are enthusiastic about your idea for change. They might have a connection to the care you’re advocating for, such as a loved one with a substance use disorder or personal experience with miscarriage. Engage green lights early. Let them draft policies, work with information technology to create charting templates, prepare staff training, create patient discharge instructions, etc. Keep an open mind: Green lights come in many forms—physicians, nurses, pharmacists, techs, navigators, security personnel, social workers, and more. All are welcome.

### Next: Make it Easy for Yellow Lights to Participate

We estimate that roughly 80% of people are “yellow lights.” These are busy people already overwhelmed by patient care, charting, keeping up with current practice guidelines, completing continuing medical education (CME) and other administrative tasks. For this group, creating change can mean more work, taking time to learn something new and disrupting an already tenuous work/life balance.

Yellow lights don’t have significant objections to your idea, but you must make it easy for them to participate. Work through as many “bumps” in the workflow as possible: Create automatic order sets; ensure clinical protocols are readily available at workstations; offer CME opportunities that provide relevant education; and share examples demonstrating improved patient care. Make the right thing the easy thing and regularly remind your peers of the change so it stays fresh.

### Motivate Red Lights…Later

Red lights are typically few but vocal in opposition. Their arguments may reflect gaps in current knowledge, reliance on outdated frameworks, or the influence of personal beliefs. Identify these people early. Meet with them to acknowledge their concerns but put them in a proverbial “parking lot” while you focus on green and yellow lights to get your change-making efforts off the ground. Keep group discussions focused and goal oriented. Later, you may need to use a senior-level leader or strategic peer pressure to bring them along. When thoughtfully engaged, red lights can flip to become some of the most productive green lights.

**Table:** CASE STUDY: FROM FRAMEWORK TO ACTION

From Framework to Action	Co-author Dr. Alicia M. Gonzalez used this approach to implement medication for opioid use disorder (MOUD) with buprenorphine at a hospital on California’s central coast.
Critical Points of Engagement for MOUD Implementation	Hospital Community Board of Directors and CEO Set strategic priorities for the hospital Hospital CMO and Chief of Staff Ensure standard of care is met by clinical teams Emergency physician and nursing leadership Care for emergency patients with OUD Case management and social work leadership Oversee staff typically consulted for patients with substance use disorder. Pharmacy Oversees hospital medication formulary and Pharmaceuticals and Therapeutics (P&T) committee
Traffic Light Approach to Engagement in Implementing MOUD	Green lights: Advocated to P&T to include buprenorphine on formulary; worked with IT to ensure correct doses were orderable in the EHR; uploaded custom patient discharge instructions into the EHR; designed and hosted physician and nursing training on buprenorphine; created partnerships with community clinics to ensure “warm hand off” for patients being started on buprenorphine in the ED to have prompt access to ongoing outpatient care; presented to the hospital board and community partners to increase awareness of the opioid epidemic and the hospital’s treatment program, established initial data-driven SMART goals and tracked progress. Yellow lights: Completed recurrent training on MOUD clinical protocols; received feedback on positive outcomes for their patients started on buprenorphine; were solicited for feedback on making the program and related workflow smoother; were engaged to assist with red lights on shift as MOUD became the clear standard of care in the ED. Red lights: Concerns were heard early on and addressed openly over time in group settings, eg via education to dispel myths or incorrect assumptions; were given time to see MOUD success cases from green and yellow lights. Six to nine months after implementation, clinicians still resistant to providing MOUD to patients were given one-on-one feedback and coaching by the medical director, with time to vocalize and address any remaining concerns but understanding practice variance was now considered outside the standard of care. Once each red light’s concerns were addressed in a private setting with clear success of the program demonstrated, no red light required performance improvement planning.
MOUD Project Timeline	Months 1–3: Green lights discovered; team of champions assembled; first meetings hosted with discussion of goals and timeline. Months 3–6: Naloxone distribution program implemented. Given early resistance to MOUD based on lack of knowledge about its efficacy and evidence, in-hand naloxone distribution was a less controversial way to engage the entire ED in combatting the opioid epidemic and raised awareness of the issue. Months 3–9: Green lights continued to work on infrastructure to ensure smooth workflows for clinical and support staff, eg, EHR orders, patient instructions, and preparing job-specific clinical training. Leaders of relevant departments (ED, Nursing, Case Management/Social Work, Pharmacy) were engaged in training timeline development. Months 6–12: Initial clinician training completed, first patients treated with MOUD from the ED. Every initial patient was followed closely, and clinicians were given feedback on successful outcomes to demonstrate the effectiveness of the treatment. Green lights provided most buprenorphine. Months 9–12: Yellow lights were given re-training, frequent reminders, and opportunities to ask questions in real time when caring for patients as well as at department meetings. Increasingly, starting buprenorphine became the standard of care for patients with OUD. Months 12–18: Red lights who still carried significant concerns were addressed one-on-one by the ED medical director. Months 12–24: Group and individual data shared with the team at specific intervals, along with reminders about clinical protocols to reinforce and expand/layer knowledge of MOUD. Education and training expanded outside the ED to medical, critical care, surgery, and labor and delivery teams throughout the hospital. Note: This work took place starting in January 2021, in part limited by significant hospital attention being focused on the volatile COVID-19 pandemic. And yet significant progress was made, and the program was able to succeed.

*CEO*, chief executive officer; *CMO*, chief medical officer; *ED*, emergency department; *EHR*, electronic health record; *OUD*, opioid use disorder; *SMART*, specific, measurable, achievable, relevant, and time-bound goals.

## SEVEN STEPS TO SUSTAINABLE CHANGE

You’ve laid the groundwork, you understand your hospital’s inner workings, and you know your key players. Next: Implement the Bridge Center’s Seven Steps for Sustainable Change.

1. *Build your team*. Build a core team of green and yellow lights and assign tasks that use their strengths. Factor in legal, hospital policy, or religious considerations specific to your work, and engage experts in these areas. Be honest about your own capabilities and bolster your team with people whose expertise complements your own. Ideally, this team should include a champion from departments you want to impact, including nursing, patient navigation, and pharmacy. Set meetings with clear action-item driven agendas and stick to them.
2. *Research local examples*. Look to academic medical centers in your area, which are often ahead of community hospitals in adopting new approaches to patient care. Find nearby hospitals already implementing your proposed change. Competition in this context can be a driver for good and can help push past an initial “no.” No leader wants their facility to be behind the times.
3. *Map action items and a realistic timeline*. Define the true north: What are you trying to see happen? Establish a project plan with a timeline and goals. This guidance might seem simple to someone with a background in project management, but that skillset is rarely taught to clinicians. Posters, brochures, and hospital marketing need to be updated. Staff need to be trained; an education plan must be created for all stakeholders, including less-considered teams such as security guards, custodial staff, hospital phone operators, and volunteers.
4. *Buckle up and facilitate*. There is no getting around it: Change-making is work and requires cross-disciplinary communication. Technology is your friend. Set reminders for yourself based on your project plan and schedule reminder emails to send in advance. If this is not your strength, engage a partner who is good at it. Someone else may have more experience with this skillset, and it can be a great opportunity to engage non-clinical champions such as patient advocates or hospital volunteers.
5. *Have a strategy for inclusion*. Engage others, focusing on what’s in it for them. Green lights bring their own passion and inner “why.” Yellow lights may respond to real examples of how this change made the workflow easier for a colleague or improved a specific patient’s outcome. Others care more about decreasing repeat emergency department (ED) visits, shortening length of stay, or improving efficiency. Even red lights care about keeping up with the standard of care and don’t want to be outliers. Focus your message on your audience.
6. *Secure executive support*. It’s important to have a senior-level leader on your team supporting this change. C-suite members, medical staff leaders, and department chairs all maintain influence in the hospital. Use your executive-level support to facilitate introductions early on and keep them engaged to help with red lights as another source of listening and acknowledgment, and affirm this change is the new expected standard.
7. *Measure and report back*. Share success back to everyone, loudly and often, in both quantitative and qualitative formats. Perhaps you have a simple graph showing an improvement in a measure of patient care alongside the story of a patient who achieved a better outcome because of this change. Show your team that this change matters and you are not wasting their time. The work is important enough to monitor, measure, and celebrate. Repeat this step again and again.

## CONCLUSION

Change-making is an iterative process, but it does not have to take as long as the literature typically claims. Empowered clinical team members, motivated by making their workplace and patient care better, can do great things if given the tools to succeed. Those tools, however, are not taught to us as standard practice in medical training. Our goal in sharing these lessons learned at Bridge is to equip every clinical care team member with the basic knowledge necessary to make positive change. The best ideas to improve patient care come from frontline clinicians; our goal is to see them come to life.
